# Influence of Mothers’ Nutrition Knowledge and Attitudes on Their Purchase Intention for Infant Cereal with No Added Sugar Claim

**DOI:** 10.3390/nu10040435

**Published:** 2018-03-30

**Authors:** Tzu-Yun Chien, Yi-Wen Chien, Jung-Su Chang, Yi Chun Chen

**Affiliations:** 1 School of Nutrition and Health Sciences, Taipei Medical University, Taipei 110, Taiwan; b506100083@tmu.edu.tw (T.-Y.C.); ychien@tmu.edu.tw(Y.-W.C.); susanchang@tmu.edu.tw (J.-S.C.); 2 Graduate Institute of Metabolism and Obesity Sciences, College of Nutrition, Taipei Medical University, Taipei 110, Taiwan

**Keywords:** sugar, no added sugar, mother, infant, nutrition knowledge, attitude, purchase intention

## Abstract

A higher sugar intake in infancy might result in a predisposition to a higher sugar intake in later childhood. In Taiwan, many commercial infant and toddler foods with nutrition claims have high sugar content. This study explored the influence of mothers’ knowledge and attitudes on their purchase intention for infant food with nutrition claims. This study was a cross-sectional survey. An online survey was distributed to 940 mothers who had a child aged between 4 months and 3 years; 40% of mothers tend to misunderstand that “no added sugar” (NAS) indicates no sugar or less sugar content and, thus, that NAS infant cereal is healthy. Approximately 50–70% of mothers believe that NAS infant cereal is more natural, healthier, and contains less sugar. Attitude toward the NAS claim was found to be a mediating variable between sugar-related knowledge and purchase intention. The lower the level of sugar-related knowledge was, the more positive the attitude toward NAS infant cereal and the higher the purchase intention for NAS infant cereal were. In the future, regulation of no added sugar is needed to avoid the misleading information and mothers’ sugar-related knowledge need to be improved through nutrition education.

## 1. Introduction

Overweight and obesity can increase the risk of many health problems, including diabetes, heart disease, and certain cancers [1,2,3]. The World Health Organization (WHO) reported that in 2015, the number of overweight children under the age of 5 was estimated to be more than 42 million globally, and almost half of all overweight children under 5 years old live in Asia [4]. Compared with the 2015 data of the World Obesity Federation, the overweight rates of children in Taiwan were the highest in Asia (boys: 26.1%, girls: 21.4%) [5]. High sugar intake is of concern because of its association with poor dietary quality and obesity [6]. The American Heart Association indicated that both adults and children weight gains over the past 30 years were related to increased sugar intake [7]. Several studies have also confirmed that children who consume more sugar have a higher obesity rate than those who consume less sugar [8,9,10].

The WHO defined sugar intake as the consumption of added sugar and natural sugar present in syrups, fruit juices, and fruit juice concentrates and recommended reducing the free sugar intake to less than 10% of total energy intake in both adults and children [11]. Notably, children in Western countries obtained approximately 16–26% of calories from sugar [12]. Two nutrition surveys in Taiwan revealed that free sugar intake from snacks and beverages contributed to >10% of calories in children’s total energy intake [13,14].

The introduction of complementary foods to infants is a critical transition in the development of their eating behavior. The taste preference for sugar is mostly innate [15]. Sugar contributes to the overall pleasure and enjoyment of food. Although genetically related individual differences exist, repeated offering of certain foods can modify innate preferences [16]. Foterek et al. observed that a higher free sugar intake in infancy might result in a predisposition to a higher free sugar intake in later childhood [17].

Commercial complementary foods are convenient for parents to include in their infants’ diet. However, two surveys in the United States found that numerous commercial infant–toddler foods, snacks, and desserts had added sugars or high sugar content [18,19]. In Taiwan, 54.4% of 363 commercial complementary foods contained >10% calories from sugar [20]. When parents purchased infant or toddler food, they considered the information on the food package, including the picture, brand, nutrition label, and claims [21,22]. Munsell et al. found that parents preferred products with claims such as “low-calorie”, “real/natural”, “vitamin C” and “antioxidants” when they purchased sugar-sweetened beverages (SSBs) for their children, even though these SSBs were also nutrient-poor [23]. An online survey found that nutrient claims on unhealthy products led parents to perceive these products to be more healthy and made them more likely to select these products [24]. The other study also found that nutrition claim on high-sugar cereals misled parents to believe these products to be a good nutritional quality and parents had greater willingness to buy them [25]. Thus, it appears that health and nutrition claims can be very misleading and parents really need to understand the nutrition labels and information well to be able to distinguish between correct claims and misleading claims.

Previous studies have reported that parents’ nutrition-related knowledge and attitudes influence their feeding behavior toward children [26,27]. Consumers who lack nutrition knowledge may be limited in their ability to understand and evaluate health claims [28]. By contrast, consumers with greater nutrition-related knowledge can use nutrition labels to avoid misunderstanding products based on health claims [29]. Andrews et al. suggested that the negative relationship between nutrition-related knowledge and motivation to buy food with nutrition claims should be strengthened [30]. A previous study in Taiwan found that compared to infant food without “no added sugar” (NAS) claims, a higher percentage of NAS infant food contained more than 10% calories from sugar [20]. Also, few studies have investigated whether mothers’ nutrition-related knowledge and attitudes affect their decision to purchase children’s food with nutrition claims. Therefore, this study investigated mothers’ sugar-related knowledge and attitudes and the influence of these factors on the mothers’ purchase intention for infant cereal with sugar-related claims.

## 2. Materials and Methods

### 2.1. Study Design and Sample

This study was a cross-sectional survey. An anonymous and voluntary online survey was conducted through SurveyMonkey. Data were collected from May 2017 to July 2017. Various parenting social networks (e.g., BabyHome, mothers’ groups on Facebook, and BabyMother on a bulletin board system) were approached, and those who agreed to distribute or advertise the study posted a link to the online questionnaire on their networks. The opportunity to win commercial vouchers (NT$200) was used as an incentive. Mothers who lived in Taiwan, spoke Chinese, had a child aged between 4 months and 3 years, were their child’s primary caregiver, and had fed their child infant cereal were eligible for the study. Those who had duplicate IP addresses and who did not finish the questionnaire were excluded. The study protocol was approved by the Taipei Medical University—Joint Institutional Review Board (N201608046). Informed consent was obtained from participants through screening questions before the completion of the main questionnaire.

In accordance with data collected in previous studies [26,27], the questionnaire comprised several parts. First, we collected the demographic characteristics of the mothers, including age (≤30, 31–34, or ≥35 years), education (≤high school, undergraduate, or ≥graduate), medical background (mother were health professional, such as medical doctors, dietitians or nurses:yes or no), parity (child was firstborn or non-firstborn), household monthly income (≤NT$50,000 or >NT$50,000), and child’s age (4–12 months, 13–24 months, or 25–36 months). The second part of the questionnaire assessed sugar-related knowledge, and the third part focused on mothers’ sugar-related attitudes and health awareness. Finally, the mothers were asked about their intention to purchase NAS infant cereal, since 50% of infant cereals in Taiwan were with NAS claim [20].

The pilot study included 32 mothers who had a child aged between 4 months and 3 years. Survey items were tested for consistency, comprehensibility, and ambiguity. Five child nutrition and statistical professionals reviewed and revised the questionnaire. Cronbach’s α was performed to assess the intercoder reliability. The generally accepted lower limit for Cronbach’s α value is 0.600 [31]. Final Cronbach’s α values for each subscale of the questionnaire were in the range of0.627–0.884 (attitude toward NAS infant cereal = 0.803; importance of sugar content = 0.627; trust in package information = 0.738; health awareness = 0.884; purchase intention for NAS infant cereal = 0.859).

### 2.2. Measures

The dependent variable of purchase intention for NAS infant cereal was measured using two statements: “It is highly likely that I will purchase NAS infant cereal” (1 = highly unlikely; 5 = highly likely) and “I would recommend NAS infant cereal to my friends and family” (1 = highly unlikely; 5 = highly likely). The total score of purchase intention was between 2 and 10 points.

The independent variables that were potential predictors included demographic characteristics and mothers’ sugar-related knowledge, sugar-related attitudes, and health awareness. Sugar-related knowledge included statements on “sugar and health,” “sugar and food,” “infant cereal and nutrition,” and “sugar intake.” Mothers were asked to determine whether the statements were correct, with higher scores indicating a higher knowledge level. The total knowledge scores were between 0 and 12 points. Sugar-related attitudes included statements on “attitude toward NAS infant cereal,” “importance of sugar content,” “trust in package information,” and “Perception of nutrition label,” and were rated using a 5-point Likert scale ranging from “strongly agree” to “strongly disagree,” with scores in the range of 4–20, 2–10, 2–10, and 1–5, respectively. Health awareness was measured by five questions based on the same 5-point Likert scale, providing a total score between 5 and 25 points [32].

### 2.3. Statistical Analysis

Data were analyzed using SPSS version 18.0 and the PROCESS add-on developed by Hayes [33]. Descriptive statistics were used for analyzing participants’ demographic characteristics and scores on the health awareness, knowledge, attitude, and purchase intention scales. One-way analysis of variance (ANOVA) and an independent-sample *t* test were used to compare scores on sugar-related knowledge, sugar-related attitude, health awareness, and purchase intention scales across participant demographic characteristics, followed by a Scheffe post hoc test. The correlations between mothers’ sugar-related knowledge, sugar-related attitude, health awareness, and purchase intention were tested using the Pearson product–moment correlation coefficient. Multiple linear regressions of intention on knowledge, attitude, and health awareness were performed to identify significant predictors. Bootstrapping was used to test the significance of the indirect effects of knowledge on purchase intention in a mediation model [34]. Statistical significance was set at *p* < 0.05.

## 3. Results

### 3.1. Participant Characteristics

Of the 1535 participants who answered the online questionnaires, 940 were considered eligible and 595 were excluded because they had no experience of using infant cereal, no children aged 4 months to 3 years, or did not complete the questionnaire. Table 1 presents the demographic characteristics of the sample population.

The majority (70.7%) of the participants were ≥31 years old. Most of those who completed the questionnaire were educated at university level or above (92.6%) and did not have a medical background (81.9%). Nearly two-thirds of the participants had a household income of more than NT$50,000.

### 3.2. Sugar-Related Knowledge

Table 2 presents the correct rate of mothers’ sugar-related knowledge. The total average correct rate was 61.2%. The “sugar and food” and “sugar intake” sections had the lowest correct rates. Most participants knew that excessive free sugar intake is harmful to children’s health. In the “sugar and food” section, 93% of the mothers were unaware that natural fruit sugar is not healthier than fructose added during processing, 40% considered NAS to signify that the product is sugar free, and 74.9% did not know that sweetness is derived from ingredients aside from added sugar. In the “sugar intake” section, 78.1% of the mothers did not know that daily free sugar intake should be less than 10% of total calories, and half of them did not know that carbohydrates are different from sugar.

### 3.3. Sugar-Related Attitude

Table 3 presents the mothers’ attitudes toward sugar-related information. More than half of the mothers had a positive attitude toward NAS infant cereal. Compared with other infant cereals, 74.0% of the mothers believed that NAS infant cereal contained less sugar, and 70.9% of them believed that it was healthier. Most of the mothers also believed that noting the sugar content in a product was important. However, only half of the mothers believed the information on the package, and only 38.9% of them trusted nutrition claims. Only 25% of the mothers considered nutrition labels easy to understand.

### 3.4. Knowledge, Attitude, Health Awareness, and Purchase Intention by Demographic Characteristics

Table 4 presents the scores on sugar-related knowledge, attitudes, health awareness, and purchase intention across demographic characteristics. Mothers who had graduate degrees or above and had a medical background and household income of more than NT$50,000 had significantly higher scores in sugar-related knowledge. Mothers who were older, had a higher education level and had only one child were significantly more likely to agree that nutrition labels were easy to understand than the others. The mothers’ health awareness in our study was high (21.05 ± 2.70; total score = 25). The health awareness of mothers with a medical background (21.62 ± 2.60) was significantly higher than the those who without such background (20.92 ± 2.71). Mothers’ purchase intention for NAS infant cereal was high (7.59 ± 1.43; total score = 10), and no differences in mothers’ purchase intention were observed among different demographic characteristics.

### 3.5. Factors Related to Purchase Intention

Table 5 presents the correlations among sugar-related knowledge, sugar-related attitude, health awareness, and purchase intention. Significant negative correlations were observed between “sugar-related knowledge” and “attitude toward NAS infant cereal” (−0.152) and between “sugar-related knowledge” and “purchase intention” (−0.083), however, both of them are weak. A significant positive correlation was observed between “attitude toward NAS infant cereal” and “purchase intention,” between “importance of sugar content” and “purchase intention,” between “trust in package information” and “purchase intention” and between “health awareness” and “purchase intention”.

Table 6 presents the results of the multiple regressions for predicting mothers’ purchase intention for NAS infant cereal. After adjustment for demographic characteristics, the model explained 34.6% of the purchase intentions. Mothers with a more positive attitude toward NAS infant cereal exhibited a higher degree of trust in package information, considered sugar content in products to be important, had a higher level of health awareness, and had a higher purchase intention for NAS infant cereal.

### 3.6. Mediating Variable Between Knowledge and Purchase Intention

**Figure 1:**
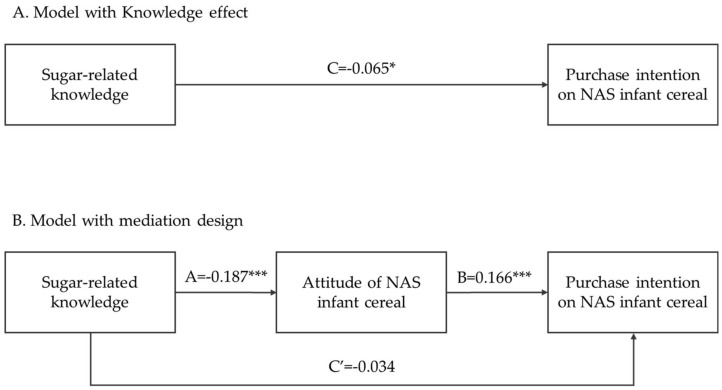
Mediating variable in the relationship between sugar-related knowledge and purchase intention of mothers with a child aged between 4 months and 3 years. Regression coefficient for (A) C: the direct affect model for the association between sugar-related knowledge and purchase intention on NAS infant cereal and (B) the mediator model accounting for attitude of NAS infant cereal. A: the association between sugar-related knowledge and attitude of NAS infant cereal; B: the association between between attitude of NAS infant cereal and purchase intention on NAS infant cereal ; C′: the effect of sugar-related knowledge on purchase intention adjusting for attitude of NAS infant cereal.

The multiple regressions provided no evidence that sugar-related knowledge can significantly predict purchase intention (Table 6). Figure 1 presents the mediating model among the variables; after ajusted age, education, medical background, parity, and household income, the direct effect of sugar-related knowledge on purchase intention (C′) was not significant (model B). However, the effects of sugar-related knowledge on attitude toward NAS infant cereal was significant. When the mediating variable (attitude toward NAS infant cereal) was considered, sugar-related knowledge had no significant direct effect on purchase intention (C′ = −0.034). Accordingly, attitude toward NAS infant cereal was observed to mediate the relation between knowledge and purchase intention in this context. The effect of knowledge on purchase intention was evident only through the variable attitude toward NAS infant cereal (B = 0.166 ***). The presence of the variable affected the relationship between sugar-related knowledge and purchase intention. Mothers’ sugar-related knowledge had a significant negative effect on attitude toward NAS infant cereal (A = −0.187 ***), which in turn had a significant positive effect on purchase intention for NAS infant cereal.

## 4. Discussion

The present study determined that “attitude toward NAS infant cereal” is a crucial predictor of mothers’ purchase intention for NAS infant cereal. This finding is consistent with those of several other studies. Harris et al. found that parents believed that cereals with nutrition-related claims were more nutritious overall and might provide specific health-related benefits for their children; these beliefs predicted a greater willingness to buy the cereal [25]. Dixon et al. also indicated that nutrient claims on the labels of energy-dense and nutrient-poor children’s foods led parents to perceive these products to be more nutritious, thereby increasing their purchase intention [24]. Munsell et al. reported that the nutrition and health claims on SSB packaging were important for predicting parents’ purchase decisions for children’s sugary drinks [23]. Other studies have also found that consumers have a “positivity bias” toward products with nutrition claims, which indicates that consumers provide a higher estimation of the product in general [35,36]. These findings provide evidence that health- and nutrition-related claims increase the perception of the healthiness of products and purchase intention for products among parents and consumers, irrespective of the real nutritional quality of these food products or SSBs.

Our study demonstrated a significant negative correlation between the mothers’ sugar-related knowledge and attitude toward NAS infant cereal. Miller and Cassady observed that people’s nutrition knowledge affected their attitude toward nutrition and health claims, in one of the few investigations on this topic [37]. In our study, 40% of the mothers misunderstood NAS to mean “sugar free,” and 50–70% of the mothers had the incorrect expectation that NAS infant cereal is more natural, healthy, and has less sugar content compared with other infant cereals. However, NAS infant cereal or other infant food products with nutrition claims may actually contain more sugar than other infant foods. A previous study in Taiwan found that 72.9% of NAS infant food contained more than 10% calories from sugar; this might be because some infant food such ascereal or pure fruit puree contains natural sugar [20]. Several studies have also reported that many infant and toddler foods with nutrition claims also contain high sugar content or less healthy than those without claims [38,39,40]. In the United States, more than half of children’s food products with health or nutrition claims were found to contain more than 20% calories from sugar [38]. Lapierre et al. reported that in the southeastern United States, children’s foods with the most nutrition and child-friendly claims had the highest sugar content [39]. In Brazil, half of children’s foods had nutrition claims, and 74.1% of these were classified as being “less healthy” [40]. Therefore, a product with nutrition claims is not necessarily healthy for children. Another study reported that women and people with children exhibited a higher interest in nutrition claims than people without children [41]. If mothers with young children are misled, a negative impact on the children’s health may result.

Previous studies have reported that in Taiwan, children’s free sugar intake from snacks and beverages is higher than the intake recommended by the WHO [13,14]. These findings indicate that parents and caregivers in Taiwan provide children with high amounts of sweet foods and beverages. Our study demonstrated an indirect effect between sugar-related knowledge and purchase intention. Through the mothers’ attitudes toward NAS infant cereal, sugar-related knowledge has a significant negative effect on purchase intention for NAS infant cereal. This observation is in concordance with a previous study, which found that knowledge affected purchase behavior by influencing attitude [42]. A previous study reported that mothers’ nutrition knowledge affects their feeding behavior [26]. Our study revealed that mothers with poor sugar-related knowledge misunderstood the NAS claim, potentially leading to unhealthy choices for children’s food and excessive sugar consumption by their children. A study confirmed that knowledge about the adverse effects of SSB was negatively associated with SSB intake among adults [43]. Another study found that parental knowledge about the American Academy of Pediatrics guidelines on juice was negatively associated with their children’s consumption of juice and SSBs [27]. Improving parental knowledge was demonstrated to reduce the consumption of SSBs among young children [44]. Our study confirmed that mothers with higher sugar-related knowledge had a lower intention to purchase NAS infant cereal.

Our study determined that the accuracy rate of knowledge on “sugar and food” and “sugar intake” among the mothers were lower than those of the other items. More than 90% of the mothers believed that natural fruit sugar is healthier than fructose added during processing. A previous study found that consumers perceive a breakfast cereal labeled as containing “fruit sugar” to be healthier than one labeled as containing “sugar” [45]. An online survey in the United Kingdom also reported that many consumers (69–89%) misclassify fruit juice as natural sugars and do not avoid these sugars in their daily diet [46]. In our study, 74.9% of mothers believed that the sweetness in infant cereal was derived from sugar added during processing and not the ingredients themselves. However, a part of the sweetness is in fact derived from the ingredients, such as the sugar in fruit. Approximately 80% of the mothers in this study did not know that free sugar intake should be less than 10% of total energy intake. This result is similar to that obtained in the UK study [46]. However, in our study, most of the mothers knew that excessive free sugar intake by infants may lead to health problems. Yet mothers may not know how to determine the amount of sugar in products, because 53.7% of them did not know the difference between sugar and carbohydrates. Future research should explore strategies to improve mothers’ knowledge of sugar through education, particularly to clarify their understanding of the healthiness and classification of sugar.

A previous study in Taiwan found that 81% of advertisements for complementary food products contained claims concerning health function or nutrient content [47]. However, in our study, the mothers had incorrect expectations of the NAS nutrition claim on children’s food packaging. Previous studies have also found that many parents have a positive attitude toward products with nutrition claims and consider these to be healthier than products without such claims [23,24,25]. In the United States, to prevent such claims from misleading parents or caregivers when they attempt to make healthy choices for their children, no nutritional content claims can be made on the food products intended for use for infants and children younger than 2 years [48]. In the European Union, to prevent consumers from misunderstanding that NAS indicates “less sugar” or “sugar free,” if sugar is naturally present in the food, the claim “contains naturally occurring sugar” should appear on the label [49]. In Taiwan, there is no rule for NAS claims and no particular standard for other nutrition claims on infant and young children’s food. However, a study in Taiwan found that a high percentage of NAS infant food contained more than 10% sugar content [20]. Our study found that a high percentage of mothers have incorrect expectations of NAS infant cereal, which could increase their purchase intention. To reduce the chances of caregivers being misled by nutrition claims, in addition to improving nutrition education, the government should modify or enhance the regulation of claims on infant food packaging.

Dixon et al. indicated that parents who read nutrition labels when purchasing children’s food are less likely to make unhealthy food choices than those who do not [24]. In our study, only 25% of the mothers considered nutrition labels easy to understand. The other study also reported similar finding that parents thought nutrition information on food package is too much and too technical [50]. A qualitative study in New Zealand found that because parents found nutrition labels difficult to read and interpret owing to the technical presentation forms, they used the list of ingredients to make food choices for their children [22]. Wills at al. also reported that food labeling is important for consumer to when deciding to buy a certain food or beverage [51]. Some studies indicated that the front of packaged system of foods could assist consumers to make healthier food choices, for its placement on the front of the package and to the design characteristics, especially color-coded Multiple Traffic Light labels have found that helping consumers easy to understand the nutrient content level, which could positively influence consumers’ choices for healthier food [52,53]. In addition to improving parents’ knowledge of nutrition labels and their ability to use it, further research is warranted to develop simple nutrition labels, such as color-coded or healthy logos, to help consumers and parents to understand the nutrient content level and to make healthy food choices.

The present study has some limitations. First, it was based on a convenient sample of mothers and was therefore not representative of the general population. Second, the proportion of mothers with a high degree of education was overrepresented. Notably, despite the group being relatively highly educated, the mothers had low scores about “sugar and food” and “sugar intake”. Also, after sugar-related knowledge, attitudes, and health awareness, then asking mother’s purchase intention might lead to mother tend to increase their purchase intention. Finally, because the survey was conducted online through social network websites, it may have failed to reach minority groups in terms of both education and income. Thus, these results possibly represent the most favorable circumstances, because less educated mothers may have an even poorer understanding of sugar and nutrition information on food packaging. These results affirm the need for increased regulations of labels on infant and children’s food in Taiwan to protect parents from the potentially misleading information conveyed by nutrition claims.

## 5. Conclusions

The common use of nutrition claims on infant and children’s food raises crucial public health concerns. We found that mothers with poor sugar-related knowledge may have inaccurate attitudes and expectations toward the nutrition claims of infant food and, thus, have higher purchase intention for NAS food. This may lead them to choose less healthy foods, resulting in their children having high sugar intake. These results affirm the need for increased regulations in Taiwan to protect consumers from the potentially misleading information conveyed by nutrition and other claims on infant food packaging. Also, mothers’ sugar-related knowledge needs to be improved through nutrition education.

## Figures and Tables

**Table 1:** Demographic characteristics of mothers with a child aged between 4 months and 3 years ^1^.

	*N*	%
Total	940	100.0
Age (years)		
≤30	275	29.3
31–34	381	40.5
≥35	284	30.2
Education		
≤High school	70	7.4
Undergraduate	687	73.1
≥Graduate	183	19.5
Medical background		
No	770	81.9
Yes	170	18.1
Parity		
First	587	62.4
Not first	353	37.6
Income, NT$/month		
≤50,000	340	36.2
>50,000	600	63.8
Child’s age, months		
4–12	438	46.6
13–24	344	36.6
25–36	158	16.8

^1^ Data are presented as numbers, percentages.

**Table 2:** Sugar-related knowledge and the correct rate of mothers with a child aged between 4 months and 3 years.

Section Statement	Answer	Correct *N* (%)	Rank	Average Correct Rate ^1^
**Sugar and health**				
Excessive sugar intake increases the future risk of obesity in infants	T	900 (95.7)	1	94.3
High sugar intake increases the risk of tooth decay in infants	T	873 (92.9)	2	
**Sugar and food**				
Natural fruit sugar is healthier than fructose added during processing	F	66 (7.0)	12	42.4
Packaging marked with NAS signifies that the product is sugar free	F	564 (60.0)	7	
If infant cereal does not taste sweet, it does not contain sugar	F	729 (77.6)	5	
The sweetness in infant cereal is derived from sugar added during processing and not the ingredients themselves	F	236 (25.1)	10	
**Infant cereal and nutrition**				
The nutritional content of infant cereal is greater than that of breast milk or infant formulas	F	829 (88.2)	3	77.0
The sooner infant cereal is added to infants’ diet, the more they will be able to obtain sufficient nutrient intake	F	723 (76.9)	6	
Starting infant cereal earlier means being able to wean the infant earlier	F	785 (83.5)	4	
Infant cereal is mostly composed of carbohydrates	T	559 (59.5)	8	
**Sugar intake**				
The carbohydrate content on a nutrition label is equivalent to the sugar content	F	435 (46.3)	9	34.1
Calories from daily sugar intake should be greater than 10% of total calories	F	206 (21.9)	11	
**Total average**				61.2

^1^ Data are presented as percentages, *N* = 940. T: true; F: flase.

**Table 3:** Sugar-related attitude and the average score of mothers with a child aged between 4 months and 3 years ^1^.

Section Statement	Agree	Neutral	Disagree
**Attitude toward NAS infant cereal**			
Infant cereal with NAS marked on the packaging is healthier than other types	666 (70.9)	190 (20.2)	84 (8.9)
Infant cereal with NAS marked on the packaging is more natural than other types	510 (54.3)	255 (27.1)	175 (18.6)
Infant cereal with NAS marked on the packaging has lower sugar content than other types	696 (74.0)	157 (16.7)	87 (9.3)
I believe that feeding my babies infant cereal with NAS can lower their future risk of obesity	474 (50.4)	300 (31.9)	166 (17.7)
**Importance of sugar content**			
Sugar should not be added to infant cereal	716 (76.2)	196 (20.9)	28 (3.0)
When purchasing infant cereal, it is crucial to pay attention to sugar content	779 (82.9)	149 (15.9)	12 (1.3)
**Trust in package information**			
I believe that the nutrition labels on infant cereal packaging are trustworthy	530 (56.4)	342 (36.4)	68 (7.2)
I believe that nutrition claims such as “no added sugar” are trustworthy	366 (38.9)	423 (45.0)	151 (16.1)
**Perception of nutrition label**			
I find the nutrition label on infant cereal packaging easy to understand	235 (25.0)	405 (43.1)	300 (31.9)

^1^ Data are presented as number(percentages), *N* = 940. NAS (no added sugar).

**Table 4:** Sugar-related knowledge, attitude, health awareness, and purchase intentions by demographic characteristics of mothers with a child aged between 4 months and 3 years ^1^.

	Sugar-Related Knowledge	Sugar-Related Attitude				Health Awareness	Purchase Intention
		Attitude Toward NAS Infant Cereal	Importance of Sugar Content	Trust in Package Information	Perception of Nutrition Label		
Total score	12	20	10	10	5	25	10
All Age (years)	7.35 ± 1.99	14.35 ± 2.74	8.19 ± 1.31	6.82 ± 1.44	2.88 ± 0.89	21.05 ± 2.70	7.59 ± 1.43
<30	7.18 ± 1.97	14.26 ± 2.66	8.05 ± 1.39	6.78 ± 1.40	3.02 ± 0.86 ^a^	21.10 ± 2.75	7.59 ± 1.39
31–34	7.39 ± 1.98	14.39 ± 2.76	8.22 ± 1.30	6.85 ± 1.44	2.82 ± 0.91 ^b^	20.93 ± 2.68	7.55 ± 1.47
>35	7.45 ± 2.02	14.37 ± 2.80	8.29 ± 1.22	6.80 ± 1.47	2.82 ± 0.89 ^b^	21.17 ± 2.70	7.63 ± 1.40
Education							
≤High school	5.97 ± 2.32 ^c^	13.93 ± 3.06	8.01 ± 1.54	6.64 ± 1.55	2.49 ± 0.78 ^c^	20.46 ± 2.85	7.74 ± 1.34
Undergraduate	7.31 ± 1.95 ^b^	14.42 ± 2.71	8.20 ± 1.29	6.80 ± 1.42	2.86 ± 0.88 ^b^	21.09 ± 2.65	7.61 ± 1.44
≥Graduate	8.02 ± 1.69 ^a^	14.24 ± 2.72	8.24 ± 1.28	6.94 ± 1.43	3.09 ± 0.94 ^a^	21.14 ± 2.81	7.43 ± 1.41
Medical background							
No	7.18 ± 2.00 ^b^	14.40 ± 2.72	8.17 ± 1.32	6.85 ± 1.45	2.86 ± 0.88	20.92 ± 2.71 ^b^	7.52 ± 1.47
Yes	8.08 ± 1.77 ^a^	14.11 ± 2.83	8.28 ± 1.23	6.67 ± 1.34	2.94 ± 0.93	21.62 ± 2.60 ^a^	7.60 ± 1.42
Parity							
First	7.29 ± 2.04	14.31 ± 2.74	8.20 ± 1.33	6.83 ± 1.46	2.94 ± 0.91 ^a^	20.99 ± 2.78	7.60 ± 1.43
Not first	7.44 ± 1.90	14.40 ± 2.74	8.17 ± 1.28	6.79 ± 1.40	2.77 ± 0.86 ^b^	21.15 ± 2.57	7.57 ± 1.43
Income							
≤50,000	7.00 ± 1.99 ^b^	14.44 ± 2.73	8.19 ± 1.31	6.82 ± 1.46	2.84 ± 0.87	21.10 ± 2.73	7.70 ± 1.41
>50,000	7.54 ± 1.96 ^a^	14.29 ± 2.75	8.19 ± 1.31	6.82 ± 1.42	2.90 ± 0.91	21.03 ± 2.69	7.52 ± 1.43

^1^ Data are presented as mean ± standard deviation, *N* = 940. ^a,b,c^ Means within each column followed by the same letter are not significantly different at the 5% level according to ANOVA with the Scheffe test or independent-sample test.

**Table 5:** Correlation between sugar-related knowledge, attitude, health awareness and the purchase intention for NAS infant cereal of mothers with a child aged between 4 months and 3 years.

	(1)	(2)	(3)	(4)	(5)	(6)	(7)
(1) Sugar-related knowledge	1						
(2) Attitude toward NAS infant cereal	−0.152 **	1					
(3) Importance of sugar content	0.164 **	0.201 **	1				
(4) Trust in package information	−0.118 **	0.477 **	0.106 **	1			
(5) Perception of nutrition label	0.182 **	0.003	−0.066	0.091 **	1		
(6) Health awareness	0.140 **	0.104 **	0.293 **	0.046	−0.067	1	
(7) Purchase intention	−0.083 *	0.492 **	0.359 **	0.406 **	0.006	0.200 **	1

* *p* <0.05, ** *p* <0.01, by Pearson correlation coefficient ; these values indicate significant differences; (1) represent sugar-related knowledge; (1) represent sugar-related knowledge; (2), (3), (4), (5) represent sugar-related attitude; (6) represent health awareness; (7) represent purchase intention.

**Table 6:** Multiple regressions to predict the purchase intentions for NAS infant cereal of of mothers with a child aged between 4 months and 3 years.

Independent Variable	Adjusted *R* ^2^	β	95% CI	*P*
	0.346	0.570	(−0.380 to 1.520)	0.239
Sugar-related knowledge		−0.036	(−0.079 to 0.006)	0.095
Sugar-related attitude				
Attitude toward NAS infant cereal		0.168	(0.136 to 0.201)	<0.001 *
Importance of sugar content		0.284	(0.221 to 0.347)	<0.001 *
Trust in package information		0.213	(0.153 to 0.274)	<0.001 *
Perception of nutrition label		0.036	(−0.053 to 0.125)	0.426
Health awareness		0.051	(0.021 to 0.081)	0.001 *

Dependent variable: purchase intention for NAS infant cereal. Adjusted for age, education, medical background, parity, and household income. * *p* <0.05 indicates significant difference, CI: confidence interval.

## Supplementary Materials

Supplementary File 1
