# Supplementary figures and images for: Influence of Mothers’ Nutrition Knowledge and Attitudes on Their Purchase Intention for Infant Cereal with No Added Sugar Claim
Source: Nutrients. 2018 Mar 30;10(4):435. doi: 10.3390/nu10040435 (PMC5946220; doi:10.3390/nu10040435)

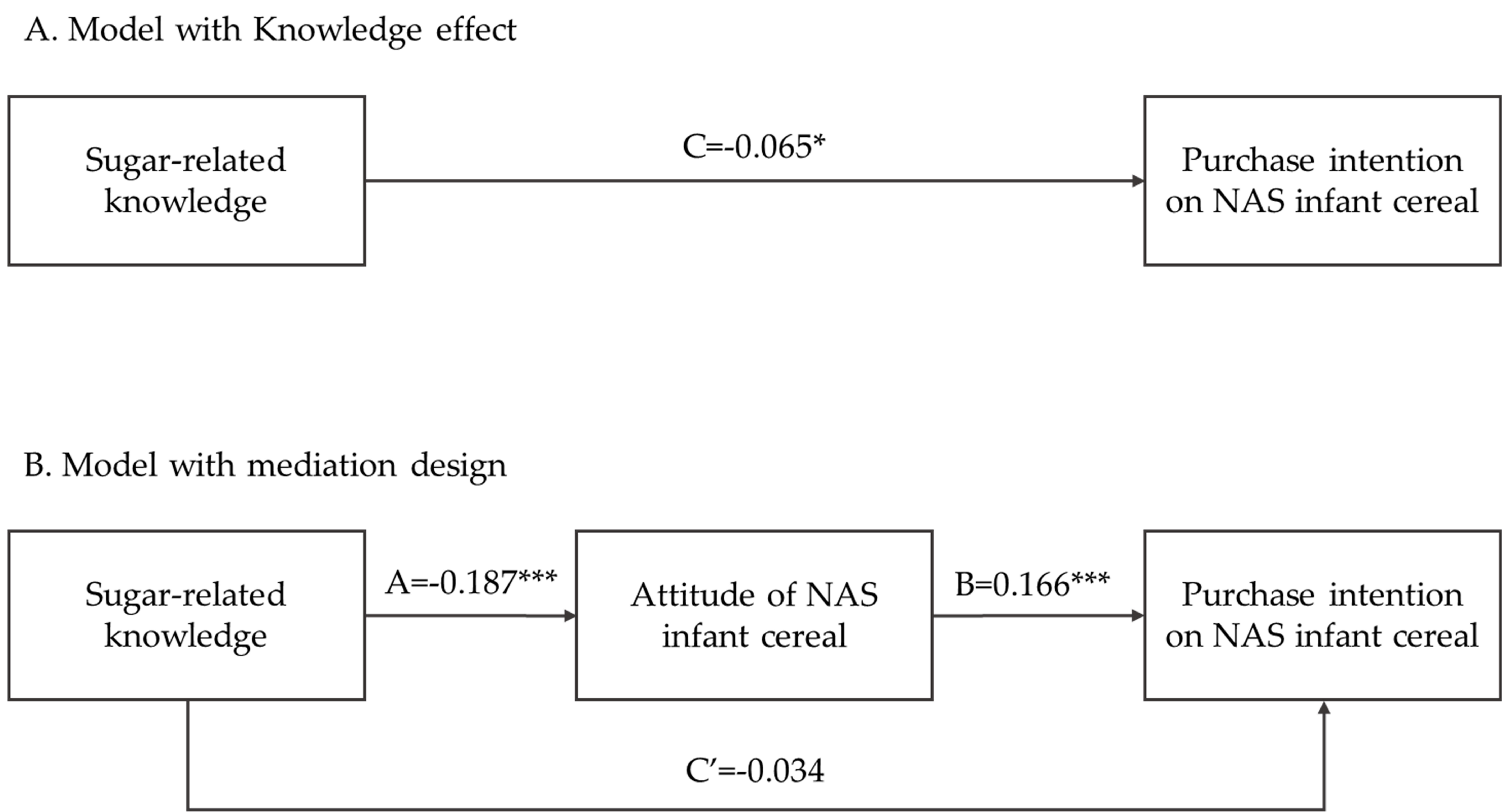

Supplement: Supplementary File 1 [file nutrients-10-00435-s001.jpg]
